# Sex, population origin, age and average digit length as predictors of digit ratio in three large world populations

**DOI:** 10.1038/s41598-021-87394-6

**Published:** 2021-04-14

**Authors:** Marina Butovskaya, Valentina Burkova, Yulia Apalkova, Daria Dronova, Victoria Rostovtseva, Dmitriy Karelin, Ruzan Mkrtchyan, Marina Negasheva, Valery Batsevich

**Affiliations:** 1 Institute of Ethnology and Anthropology, Russian Academy of Sciences, Leninsky pr., 32a, Moscow, Russia 119991; 2 National Research University Higher School of Economics, Moscow, Russia; 3 Russian State University for the Humanities, Moscow, Russia; 4 Institute of Geography, RAS, Moscow, Russia; 5 Yerevan State University, Yerevan, Armenia; 6 Moscow State University, Moscow, Russia

**Keywords:** Biological anthropology, Social anthropology

## Abstract

Recently, a number of authors have claimed that sexual dimorphism in the second-to-fourth digit ratio (2D:4D) is simply dependent on digit length and is an artifact of allometry. The goal of our study is to verify the validity of these assumptions. The study sample comprised 7,582 individuals (3,802 men and 3,780 women) from three large world populations: Europeans (n = 3043), East Africans (n = 2844), and Central Asians (n = 1695). The lengths of the second and fourth digits on both hands were measured. Digit ratios were computed according to standard procedures. Analyses were conducted separately for each hand for the whole sample and in succession for the three large populations. Additionally, we separately tested four age cohorts (≤ 13, 14–18, 19–30, and 31 ≥ years) to test the effect of developmental allometry. The second and fourth digits showed strong positive linear relationships on both hands, and demonstrated an increase with age; digit length in women from the youngest age cohort was longer or equal to that of men, and shorter than men in older age cohorts. However, the 2D:4D magnitude and its sexual dimorphism remained stable throughout the ontogeny. To test for an allometric effect on 2D:4D, the average digit lengths were calculated. Both sex and population origin were permanent reliable predictors of 2D:4D, whereas average digit length was not. Height was applied as another measure of allometric effect on the limited sample (≤ 30 years) from the European population, along with sex and age. No allometric effect was observed in this case. We conclude that sex differences in 2D:4D are not an artifact of allometry.

## Introduction

The idea for this study stemmed from two facts: 1. the high popularity of the 2D:4D ratio used for testing different traits related to androgenisation and estragenisation during critical periods of prenatal development; and 2. recently strengthened opposition against the use of the 2D:4D ratio, partly based on the idea that the 2D:4D ratio is a mere artifact of the allometric effects of digit growth. It is hypothesised that sexual dimorphism in the 2D:4D ratio is a product of the cumulative effects of both prenatal and postnatal developmental processes^1^. Hence, the 2D:4D ratio in adults may partly reflect neonatal testosterone exposure, along with prenatal exposure^1,2^. The ‘Organisational hypothesis’ suggests that prenatal sex steroids, particularly testosterone, modify growth and development in a sexually dimorphic way^3^. Due to ethical reasons, accurate measurements of prenatal testosterone exposure in humans are difficult, and a limited number of studies have been conducted in this area to date. Hence, the popularity of the use of indirect measures as a biomarker of prenatal androgenisation, namely, 2D:4D, is growing^4–7^. In the majority of studies, researchers deal with 2D:4D in postnatal samples, with a wide range of age groups^8–10^.

One of the most important questions is the extent to which the digit ratio may serve as a proxy for prenatal androgenisation, and whether 2D:4D actually indexes prenatal sex steroid exposure. Both indirect and direct data deserve mention in this respect. A recently conducted meta-analysis on the 2D:4D ratio and congenital adrenal hyperplasia (CAH) showed that the digit ratios were typically lower (i.e. more ‘male typical’) in CAH populations than in sex-matched controls^11^. This seems to provide some evidence in favour of the initial hypothesis that higher prenatal testosterone leads to the development of lower digit ratios^12^. However, Richards et al. suggested that, at least in the case of CAH, there may be a number of other possible explanations of lower 2D:4D ratios: 1. reduced concentrations of glucocorticoids and mineralocorticoids, both of which affect bone growth; 2. sex differences in the deposition of adipose tissue in the fingers^13,14^; and 3. prenatal cortisol deficiency, as well as early postnatal administration of glucocorticoids and mineralocorticoids shortly after birth in cases of CAH treatments^11^. The most recently published study reported no differences in 2D:4D in CAH and control youth samples in men and women^15^.

The data on the associations between 2D:4D and prenatal sex hormones measured from amniotic fluid and umbilical cord blood may be of some assistance^16–19^ in support of 2D:4D as a biomarker of prenatal androgenisation^11,20^. To date, such studies are rare. Malas et al. conducted a study on foetuses without pathology or malformation at 9–40 weeks of gestation, and revealed significantly higher digit ratios in female foetuses^21^. Another study, conducted on foetuses from 14 to 42 weeks^1^, revealed a slight, but still significant, sexual dimorphism in the expected direction. G. Richards reported two studies of amniotic fluid^17^ and mentioned six studies of umbilical cord blood^18^. S. Lutchmaya et al. provided evidence that prenatal sex steroids influence digit development^19^. They demonstrated that the 2D:4D ratios in two-year-old children were associated with the levels of foetal testosterone and estradiol in the amniotic fluid of their mothers in the second trimester of pregnancy. The low 2D:4D ratios were associated with high foetal testosterone in relation to estradiol. On the contrary, the high values of 2D:4D were associated with low foetal testosterone and high estradiol levels. In addition, it was found that all relationships between 2D:4D and foetal sex steroids were stronger in the right hand than in the left. However, a recent replication study examining associations between individual differences in amniotic sex hormone concentrations and digit ratio did not confirm the initial findings of Lutchmaya^22^. Hence, the hypothesis according to which a mid-trimester sex hormone concentration may affect the development of 2D:4D ratios in humans remains problematic.

Two studies by Mitsui et al. reported the level of adrenal steroid hormones in cord blood samples, and 2D:4Ds for the same individuals, measured when they became school children^23,24^. While no significant associations between prenatal androgen levels and 2D:4D were found in the first study, the second study demonstrated that 2D:4Ds (both hands) were significantly lower in males than in females (*p* < 0.01). The level of dehydroepiandrosterone (DHEA) was significantly negatively correlated with 2D:4D in males only. G. Richards noted the inconsistency of these results^18^. However, J. Manning and B. Fink, in disagreement with G. Richards, pointed to the fact that it is far from obvious ‘whether amniotic studies are the best way forward to consider links with 2D:4D’^16,21,25^, and reasoned that the ‘amniocentesis is typically performed in the second trimester (weeks 14 to 16) and cord-blood yields perinatal hormones’^16^. This suggests that amniotic studies may not be used as ‘direct’ evidence for ‘links between foetal sex hormones and 2D:4D’^16^. Hence, the problem with 2D:4D as a biomarker of prenatal androgenisation is far from resolved.

The role of androgen and oestrogen signalling in the development of sexually dimorphic digit ratios has also been investigated in animals^26,27^. It was demonstrated that androgen receptor (AR) and oestrogen receptor α (ER-α) activity were higher in digit four than in digit two, and inactivation of AR decreased the growth of digit four, whereas inactivation of ER-α increased the growth of digit four. Thus, both affect the digit ratio in mice^26^. However, these results were not confirmed in another study^28^, although the organisational morphological effects of prenatal ARs on 2D:4D have been demonstrated. The interaction effect of salivary testosterone and androgen receptor gene CAG repeats was mentioned as a potential predictor of 2D:4D in the first two years of life in males^2^. However, a recently published and more representative study with replication and meta-analysis on *AR* (CAG)n and current testosterone levels reported no significant relationships with 2D:4D at the individual level in adults^29^.

Whether 2D:4D increases after birth during ontogeny is another unresolved question. Generally, studies have suggested that the prenatal 2D:4D ratio is lower than that reported for children and adults. This means that the digit ratio increases after birth in both sexes, and the second digit grows faster than the fourth digit (positive allometric growth of digit two)^1^. Some data suggest that the 2D:4D ratio remains relatively stable during lifetimes since early childhood^4,30^, whereas other data demonstrated that some changes may take place during the prenatal period, during the first two years after birth, and during later individual life^1,2,21,31–33^. Hence, sex differences may increase from childhood to adolescence.

Population and ethnic differences in digit ratios have been reported^4,5,34–37^. In most cases, men had lower 2D:4D ratios than women from the same population. The exceptions include data reported on Yali from Papua^38^ and Hadza of Tanzania (data reported by C. L. Apicella et al.)^39^. However, the data reported by M. Butovskaya et al. on Hadza provided sexually dimorphic digit ratios in the expected direction for both children and adults^36,40^. The nature of population differences in 2D:4D, as well as differences in the degree of sexual dimorphism, is another important question for future studies.

In this general area, a group of authors^41,42^ has made a serious claim that sexual dimorphism in 2D:4D arises as an artifact of allometry. They have stated that there is no sexual dimorphism, apart from men being generally larger, and there is no need to invoke specific sex hormone effects on finger development to explain the differences between males and females. According to these authors, allometry and sexual dimorphism may be found by regressing the length of the second finger 2D (outcome variable) over the length of the fourth finger 4D (predictor variable). This view has been criticised from a methodological perspective by other scholars^43^. In particular, the ordinary least squares (OLS) regression method fails to account for ‘biological noise’, ‘natural variation’^44^, or ‘biological deviance’^45^ in the predictor variable.

In current anthropological literature, ratios have been frequently criticised in general (see, for example,^46^), mainly because ratios often fail to achieve independence of body size. However, W. Forstmeier^43^ noted that ratios, in principle, may still be independent of variation in body size. He called for the necessity of empirical testing on whether human digit ratios are independent of size, and proposed using the mean finger length [(2D + 4D)/2] as a measure of body size. J. Manning and B. Fink levelled another critique^30^. These authors have suggested that such views arise because of a misunderstanding of the nature of sexual dimorphism in digit length, and pointed to the necessity of differentiation between static and developmental allometry. J. Manning and B. Fink demonstrated that female digits in prepubertal children tend to be longer than male digit lengths, but 2D:4D is sexually dimorphic in the expected direction (males < females)^30^. After the age of 13, sexual dimorphism in digit length became progressively greater, with males exceeding that of females; however, 2D:4D has been independent of age^30^. Decades earlier, the X-ray data from the Fels study of longitudinal growth in children aged 2–18 were used by S. M. Garn et al.^47^ to demonstrate that the length of the phalanges of the digits has increased rapidly. Again, at the start, girls tended to have longer (not shorter) phalanges than boys. Around the age of 13, both sexes reached approximately equal phalange lengths^47^. Another radiologic study revealed that phalanges grew faster in boys^48^.

While phalange lengths and sex differences in phalange lengths change rapidly, the bone-to-bone length ratios are relatively stable^47–49^. That is, radiologic longitudinal, cross-sectional, and longitudinal direct finger measurements suggest that the magnitude of sex differences in 2D:4D is not linked to digit lengths. Male digit growth continues beyond the age of 18, long after the digits of females cease to grow. From the age of 20 to 30, sex differences in digit lengths are substantial, but sexual dimorphism in 2D:4D remains stable^30^.

Whether 2D:4D is a simple artefact of allometry is of great importance, given the ongoing discussion on the role of 2D:4D as a marker of prenatal androgenisation^21,33,50–53^. Furthermore, this question is important for our understanding of the data on 2D:4D and its association with a number of morphological, physiological, psychological, and behavioural traits, and life history trajectories^6,7,9,54–62^.

The goals of the current study were to test whether the sexual dimorphism in 2D:4D may be associated with allometric changes, to analyse the developmental allometric processes in prepubertal, pubertal, young adult, and older adult age cohorts, and to determine if the same model is valid for the samples from three large human populations tested in our study, namely, Europeans, East Africans, and Central Asians.

## Materials and methods

### Ethics statement

The study was conducted in accordance with the principles of the Declaration of Helsinki. The Commission for Science and Technology of Tanzania (Permits 2008–238-ER-2005–126, 2009–243-CC-2009–151, 2014–101-CC-2009–151), and the National Institute for Medical Research (NIMR/HQ/R.8a/Vol. IX/458, dated 5 September 2006) and the Scientific Council of the Institute of Ethnology and Anthropology of the Russian Academy of Sciences (protocol №1, dated 19 February 2015) approved the protocols used to recruit participants and collect data before conducting the study. All subjects provided informed verbal or written consent prior to participation. Verbal consent was deemed appropriate given the low literacy rates of our participants (in this case, consent was registered by research assistants in the presence of a particular respondent: Hadza, Datoga, Isanzu, Iraqw, and Meru). Permission for children’s participation was also obtained from their parents. The local school administrations were informed about the purpose of this study and also provided their consent.

### Participants

The present study was a cross-sectional study conducted in Russia, Armenia, and Tanzania during a number of field studies between 2004 and 2019. In total, data on 7,582 individuals (3,802 males and 3,780 females) within the age range of 4–95 years from three large world populations were collected: Europeans (n = 3043), Africans (n = 2844), and Asians (n = 1695). Europeans were represented by Russians (n = 2313), Mordva (n = 106), Ossetians (n = 364), and Armenians (n = 260); Sub-Saharan Africans were represented by East African populations from Tanzania, namely, by Hadza (n = 643), Datoga and Maasai (n = 1134), Iraqw (WaMbulu) (n = 274), and Bunty (Isanzu, Meru, and others) (n = 793); Asians were represented by Central Asian populations of Buryats (n = 606) and Tyva (n = 1089), both of Mongolian origin. The data on European populations were collected in Russia (Central Russia, Volga Region, North Ossetia-Alania) and Armenia. All Asian data were collected in Russia (Buryatia Republic and Tyva Republic). Data on Africans were collected in Tanzania (in Arusha, Manyara, and Singida Regions).

### Procedure

The data were collected by the authors of this study who are experienced in anthropometry. The second and fourth digits of participants were measured directly (with a Vernier calliper measuring to 0.01 mm) from the basal crease to the tip on both hands. Where there was a band of creases at the base of the digit, the most proximal crease was used^63^. Participants who reported injuries or deformities of the second or fourth digits were excluded from later statistical analyses. Direct measurements avoid the problem of distortion when palms are placed on a photocopier or scanner^64^. The right and left 2D:4D ratios were calculated following the procedure described by Manning et al.^12^. The repeated measures of the first and second 2D:4D for the whole sample provided an intra-class correlation of 0.94 for the right hand and 0.93 for the left. Therefore, we assumed that the differences in the between-individual measurements of 2D:4D were significantly greater than the within-individual measurement error.

We estimated the relationships between the second and the fourth digits by regressing the second digit (outcome variable) over the fourth digit (predictor variable) to reveal the proportional differences in their lengths. This was done for the whole sample, as well as for different age cohorts (see below for the divisions of such age cohorts). In addition, we revealed the age dependence of each of the two digit lengths, as well as digit ratios, by regressing these parameters over the age scales. We tested the effect sizes (Cohen’s d) of sex differences in digit ratios, as well as sex differences in mean (average) digit lengths to determine which of these parameters have a higher potential in reflecting levels of sex hormones during development.

To test for the allometric effect on 2D:4D, we used the mean (average) digit lengths, calculated as [(2D + 4D)/2], as previously reported by other scholars^40–43^. This was done because of the disproportionally fast growth of both fingers during some periods of ontogeny (particularly, the pubertal period). In addition, it may not be optimal to test the separate effect of the fourth or second digits on 2D:4D, as both fingers invest in the obtained ratio, and a simple correlation between these parameters (although in different directions) is logically expected. We plotted the digit ratio directly over the mean finger length. Next, we analysed the digit ratio as the outcome variable in a general linear model, using the GLM ANCOVA, with sex as a fixed effect and mean finger length as a covariate (main effects without interaction).

In order to test the possible differences in digit growth patterns in more detail to reveal the effect of developmental allometry (separately from static allometry), and to minimise the effect of possible noise arising from a number of reasons, including differences in life history and ageing, differences related to population origin, individual variations, and ecological and cultural-economic factors, we divided our sample into four age cohorts based on general assumptions about the periods of ontogeny: 1. prepubertal (equal and younger than 13 years); 2. pubertal (between 14 and 18 years); 3. young adults (between 19 and 30 years); and 4. older adults (aged 31 years and older). It is important to note that the fourth age cohort was underrepresented in the cases of representatives of European and Asian populations. Additionally, the sample from the European population aged 9 to 30 years was tested in separate analyses for allometric effect based on height information obtained from the same individuals. Height data were collected using a portable anthropometer.

## Results

The descriptive statistics for the 2D:4D ratio, mean finger length, and average finger length for the total sample, as well as for the three large samples are presented in Table 1. Due to various injuries, deformities, and other problems with fingers on each hand, the final reported numbers for second and fourth fingers on the right and left hands were different, and the final samples of digit ratios were: n_R2D:4D_ = 7490 and n_L2D:4D_ = 6481 (Table 1).

**Table 1:** Sex differences in finger measurements and 2D:4D ratios for both hands in total sample and European, African and Asian populations.

Parameters	Population	Sex	*N*	Mean	SD	t	df	P	95% Confidence Interval of the Difference		Cohen’s d
									Lower	Upper	
Right 2D finger	Total sample	Male	3760	67.365	7.702	13,163	7194	4.0423E−39	1.808	2.441	0.304
		Female	3756	65.240	6.211						
	European origin	Male	1445	70.650	6.544	11.935	2692	4.9027 E−32	2.126	2.962	0.437
		Female	1441	68.106	4.988						
	African origin	Male	1511	65.287	7.718	7.883	2805	4.5512 E−15	1.583	2.632	0.297
		Female	1298	63.179	6.452						
	Asian origin	Male	804	65.364	7.559	6.707	1498	2.8037 E−11	1.565	2.860	0.329
		Female	882	63.151	5.771						
Right 4D finger	Total sample	Male	3757	70.145	7.716	21.667	7136	7.1268 E−101	3.164	3.794	0.500
		Female	3755	66.666	6.109						
	European origin	Male	1441	72.633	6.679	17.743	2662	1.1514 E−66	3.420	4.270	0.645
		Female	1575	68.788	5.019						
	African origin	Male	1512	68.616	7.929	12.120	2806	5.4603 E−33	2.787	3.863	0.445
		Female	1298	65.291	6.616						
	Asian origin	Male	804	68.562	7.887	10.670	1490	1.1501 E−25	2.989	4.335	0.524
		Female	882	64.900	5.969						
Right average finger length	Total sample	Male	3740	68.728	7.593	17.536	7116	1.9764 E−67	2.468	3.090	0.405
		Female	3750	65.949	6.032						
	European origin	Male	1434	71.602	6.479	15.055	2641	3.3436 E−49	2.753	3.578	0.553
		Female	1573	68.437	4.847						
	African origin	Male	1506	66.939	7.711	10.145	2798	8.9306 E−24	2.184	3.231	0.382
		Female	1297	64.231	6.419						
	Asian origin	Male	800	66.946	6.646	8.738	1481	6.3183 E−18	2.257	3.564	0.430
		Female	880	64.035	5.775						
Right 2D:4D ratio	Total sample	Male	3740	0.961	0.037	−21.755	7489	8.4128 E−102	−0.020	−0.017	0.493
		Female	3751	0.979	0.036						
	European origin	Male	1434	0.973	0.035	−13.743	2984	1.0206 E−41	−0.020	−0.015	0.514
		Female	1573	0.991	0.035						
	African origin	Male	1506	0.952	0.038	−11.517	2801	5.0857 E−30	−0.019	−0.014	0.427
		Female	1297	0.968	0.037						
	Asian origin	Male	800	0.954	0.032	−12.725	1679	1.772 E−35	−0.023	−0.017	0.635
		Female	881	0.974	0.031						
Left 2D finger	Total sample	Male	3261	67.341	7.648	10.015	6284	1.9592 E−23	1.393	2.071	0.248
		Female	3228	65.608	6.243						
	European origin	Male	1167	70.043	6.784	9.533	2140	3.9689 E−21	1.846	2.802	0.389
		Female	1265	67.719	5.027						
	African origin	Male	1301	66.038	7.638	2.557	2370	0.011	0.177	1.346	0.105
		Female	1086	65.276	6.907						
	Asian origin	Male	806	65.531	7.768	7.506	1494	1.0407 E−13	1.872	3.197	0.368
		Female	884	62.997	5.882						
Left 4D finger	Total sample	Male	3261	69.922	7.694	16.162	6266	1.3533 E−57	2.477	3.161	0.401
		Female	3228	67.103	6.296						
	European origin	Male	1162	71.999	6.985	13.572	2133	2.6265 E−40	2.921	3.908	0.555
		Female	1264	68.585	5.189						
	African origin	Male	1294	68.706	7.834	5.284	2352	1.3838 E−7	1.027	2.240	0.217
		Female	1082	67.072	7.221						
	Asian origin	Male	805	68.880	7.882	11.352	1293	1.0502 E−28	3.196	4.531	0.560
		Female	882	65.017	5.925						
Left average finger length	Total sample	Male	3256	68.620	7.569	13.249	6245	1.5657 E−39	1.934	2.606	0.329
		Female	3225	66.350	6.160						
	European origin	Male	1159	71.006	6.777	11.724	2115	8.4438 E−31	2.378	3.333	0.480
		Female	1263	68.150	4.986						
	African origin	Male	1293	67.361	7.631	4.013	2353	0.000062	0.615	1.790	0.167
		Female	1080	66.159	6.953						
	Asian origin	Male	804	67.206	7.717	9.543	1487	5.4727 E−21	2.542	3.858	0.468
		female	882	64.006	5.819						
Left 2D:4D ratio	Total sample	male	3256	0.963	0.036	−16.684	6471	3.1941 E−61	−0.016	−0.013	0.422
		female	3225	0.978	0.035						
	European origin	Male	1159	0.974	0.034	−10.649	2379	6.7029 E−26	−0.017	−0.012	0.418
		female	1263	0.988	0.033						
	African origin	Male	1293	0.962	0.038	−7.816	2371	8.102 E−15	−0.015	−0.009	0.320
		female	1080	0.974	0.037						
	Asian origin	Male	804	0.961	0.033	−11.414	1684	4.0782E−29	−0.021	−0.015	0.562
		female	882	0.969	0.031						

Sex differences presented according to Student’s T test (*t* test statistics, *SD* std. deviation, *df* degrees of freedom, *p* statistical significance).

The same table presents information on sex differences for these traits and effect sizes (Cohen’s d). T-tests, conducted for the whole sample, as well as separately for each of the three populations, revealed significant sex differences in all tested parameters (2D, 4D, average finger length, and 2D:4D) for both hands (Table 1). The digit ratios on both hands were lower for males than for females, both for the whole sample and separately for each of the tested populations, with small to medium effect sizes.

**Figure 1:**
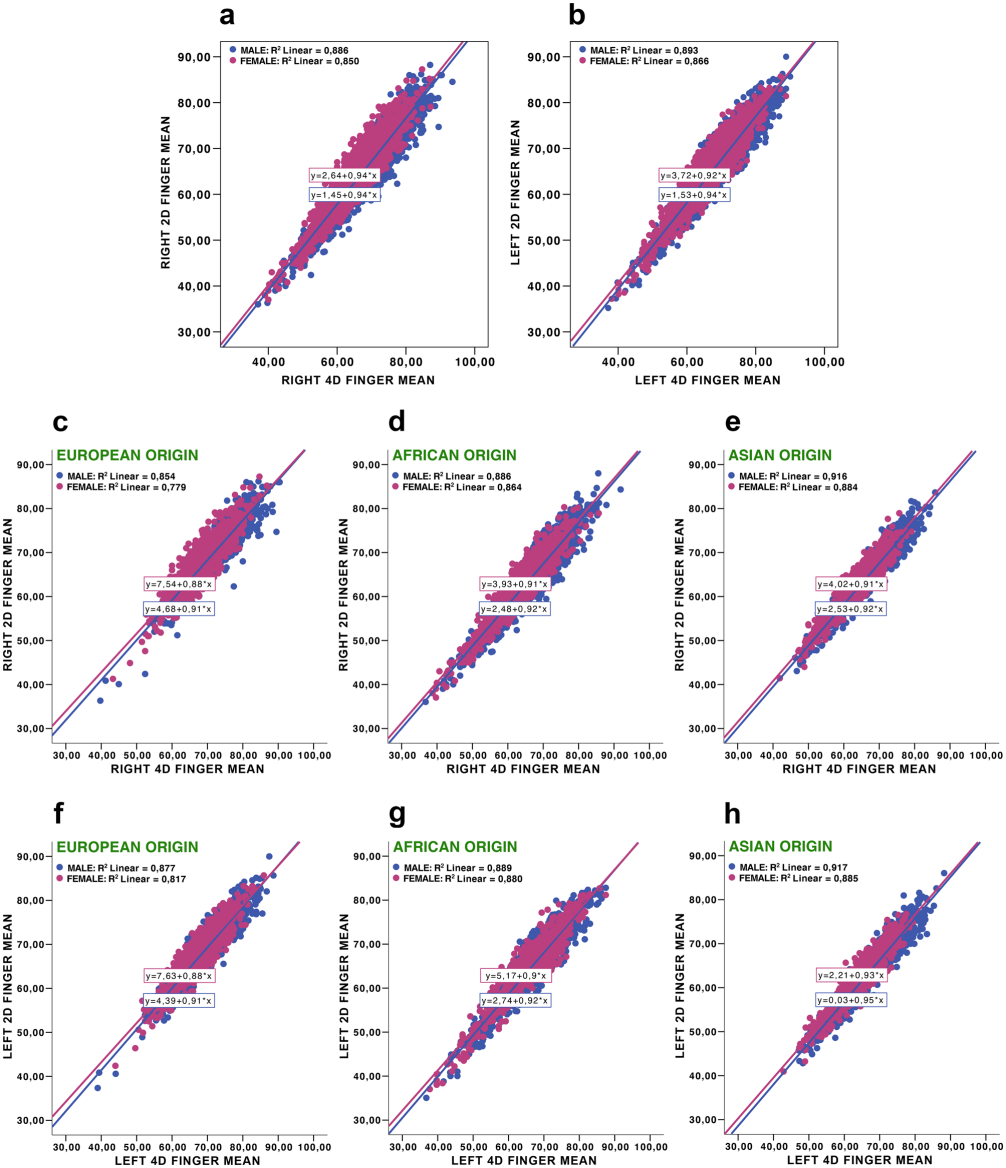
Ratio 2D finger means to 4D finger means: right 2D finger means to right 4D finger means in total sample (**a**), left 2D finger means to left 4D finger means in total sample (**b**), ratio right 2D finger means to right 4D finger means in three populations (European origin (**c**), African origin (**d**), Asian origin (**e**)), ratio left 2D finger mean to left 4D finger mean in three populations (European origin (**f**), African origin (**g**), Asian origin (**h**)).

We regressed the 2D on the 4D length for the whole sample and separately for each of the three populations (Fig. 1).

According to the one-way ANOVA, the three populations differed by digit ratios on both hands in males (right hand: F_2.3737_ = 147.242, *p* = 2.806E−62; left hand: F_2.3253_ = 96.716, *p* = 1.578E−41) and females (right hand: F_2.3748_ = 161.514, *p* = 3.934E−68; left hand: F_2.3222_ = 96.151, *p* = 2.760E−41). Right hand digit ratios in males also differed significantly between populations; 2D:4D was higher in Europeans than Africans and Asians (Post-Hoc, DunnettT3: *p* = 5.0E−6 and *p* = 0.001). The same was true for European females compared to Africans and Asians (Post-Hoc, DunnettT3: *p* = 8.0E−6 and *p* = 1.0E−6). Again, the left hand 2D:4D was higher in Europeans than African and Asian males (Post-Hoc, DunnettT3: *p* = 7.0E−6 and *p* = 0.001), and females (Post-Hoc DunnettT3: *p* = 3.0E−6 and *p* = 4.407E−7).

We conducted the GLM ANCOVA four-way analyses with 2D:4D on each hand as outcome variables, and sex and population as independent predictors, age and average finger length as covariates for the whole sample (Table 2), and GLM ANCOVA three-way analyses with sex, age, and average finger length as covariates separately for the three populations (Table 2). This was done to test the main effects of these predictors on the 2D:4D values. Sex was a significant predictor for both the right and left hands in all samples, as well as for each study population. The effect of population was significant for the whole sample in the case of the right hand (medium effect size) and the left hand (small effect size). The effects of the mean digit lengths (both hands) for the total sample and for each of the three populations were not statistically significant.

**Table 2:** The GLM ANCOVA analyses for outcome variables the right and left 2D:4D ratios and sex, population, age, average finger length for the right and left hand in the whole sample, and sex, age, average finger length for each population.

Population	Dependent variable	R^2^	Df	Independent variables	F	P	η^2^
Total sample	R2D:4D	0.140	1	Sex	445.765	3.677E−96	0.056
			2	Population	295.104	4.373E−124	0.073
			1	Age	70.237	6.225E−17	0.009
			1	R average finger length	0.571	0.450	0.000
	L2D:4D	0.096	1	Sex	282.603	4.161E−62	0.042
			2	Population	171.900	1.816E−73	0.050
			1	Age	7.301	0.007	0.001
			1	L average finger length	1.361	0.243	0.000
European origin	R2D:4D	0.060	1	Sex	178.003	1.722E−39	0.056
			1	Age	1.737	0.188	0.001
			1	R average finger length	1.137	0.286	0.000
	L2D:4D	0.045	1	Sex	103.720	7.018E−24	0.041
			1	Age	0.286	0.593	0.000
			1	L average finger length	0.285	0.593	0.000
African origin	R2D:4D	0.070	1	Sex	140.822	1.001E−31	0.048
			1	Age	69.291	1.311E−16	0.024
			1	R average finger length	1.225	0.269	0.000
	L2D:4D	0.028	1	Sex	62.874	3.363E−15	0.026
			1	Age	5.718	0.017	0.002
			1	L average finger length	0.026	0.872	0.000
Asian origin	R2D:4D	0.089	1	Sex	151.732	1.934E−33	0.083
			1	Age	0.044	0.835	0.000
			1	R average finger length	0.015	0.904	0.000
	L2D:4D	0.087	1	Sex	137.735	1.256E−30	0.076
			1	Age	5.330	0.021	0.003
			1	L average finger length	7.706	0.006	0.003

*R*^*2*^ R Squared, *df* degrees of freedom, *F* F test statistics, *p* statistical significance, *η*^*2*^ Partial Eta Squared effect size.

**Figure 2:**
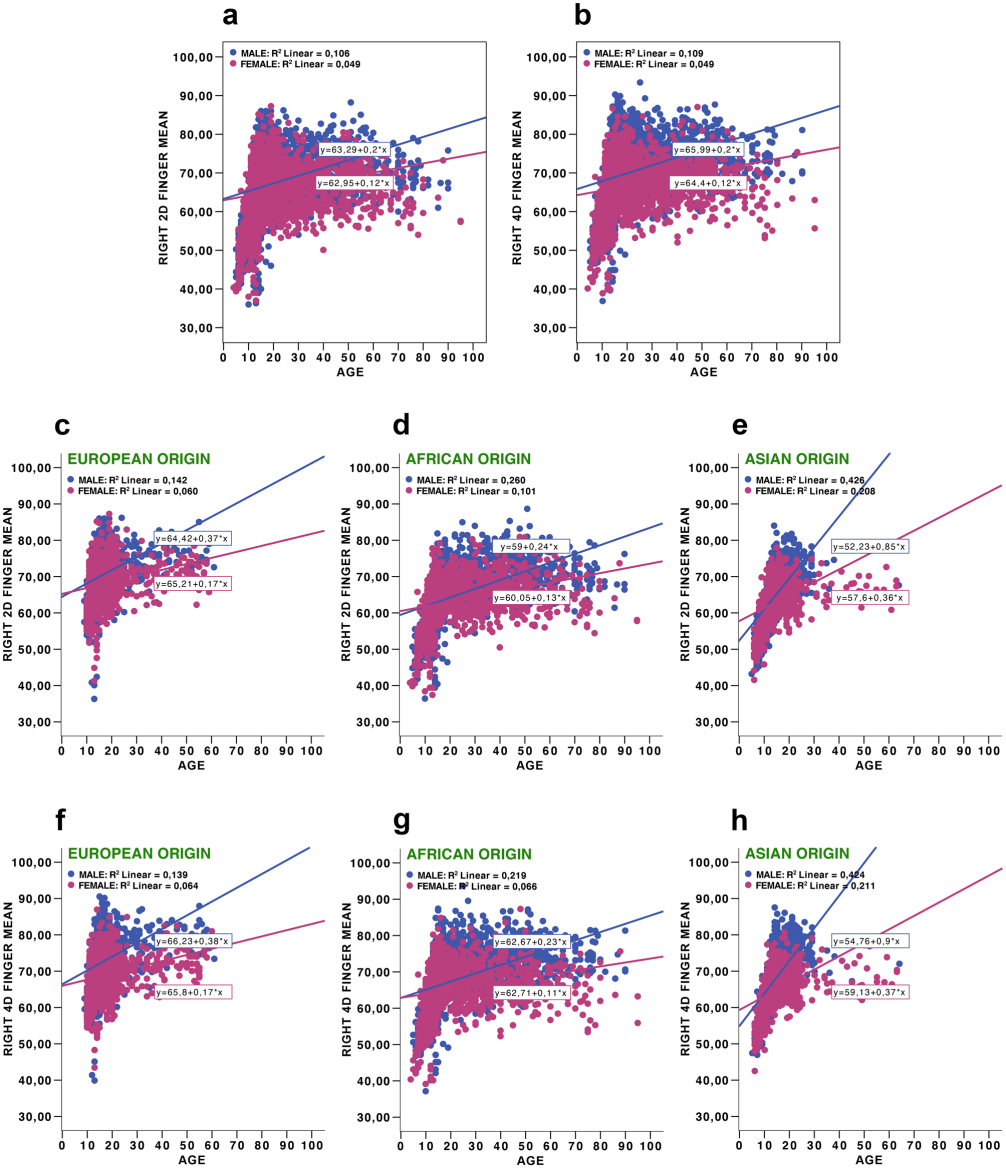
Right 2D and 4D finger means to age: right 2D finger means to age in total sample (**a**), right 4D finger means to age in total sample (**b**), right 2D finger means to age in three populations (European origin (**c**), African origin (**d**), Asian origin (**e**)), right 4D finger means to age in three populations (European origin (**f**), African origin (**g**), Asian origin (**h**)).

**Figure 3:**
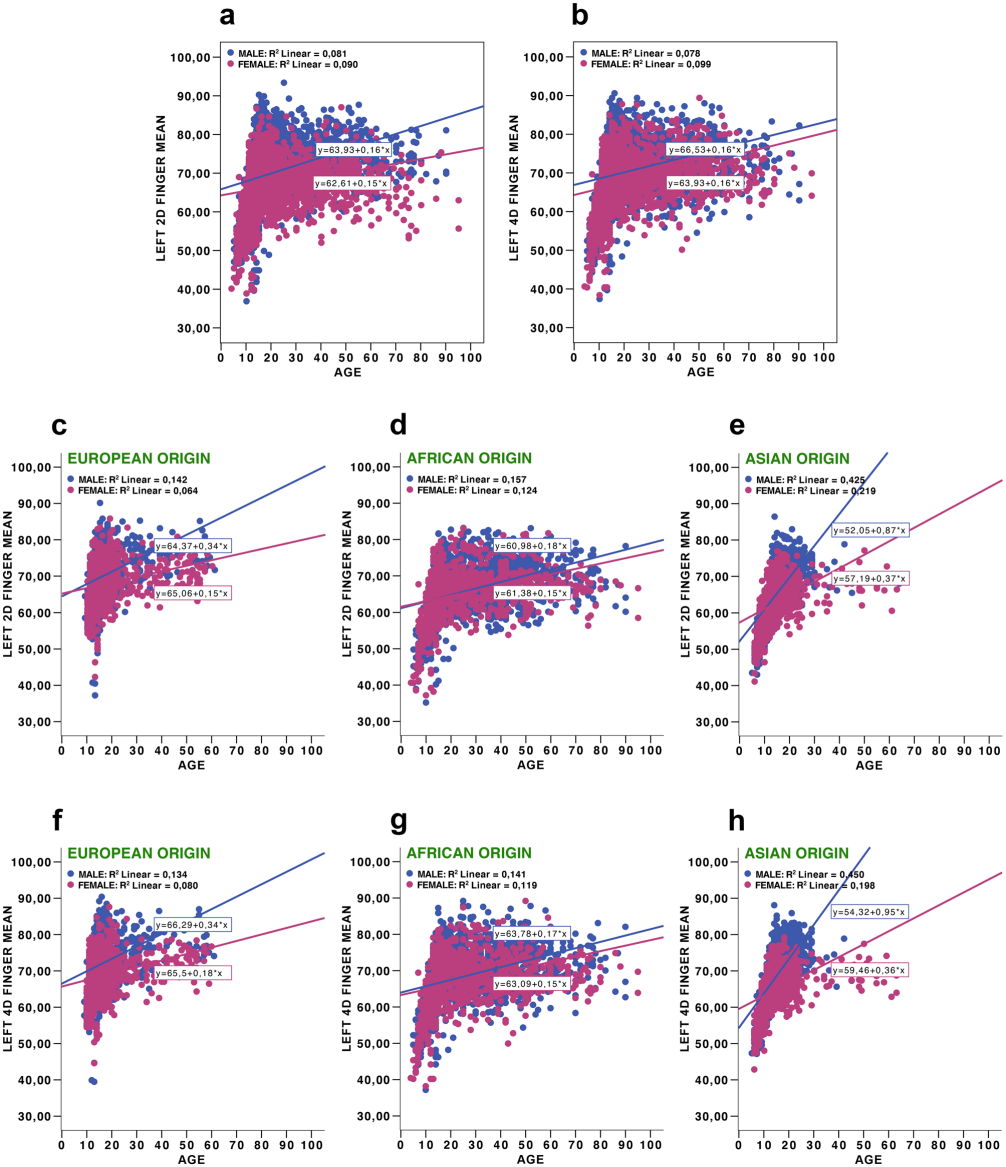
Left 2D and 4D finger means to age: left 2D finger means to age in total sample (**a**), left 4D finger means to age in total sample (**b**), left 2D finger means to age in three populations (European origin (**c**), African origin (**d**), Asian origin (**e**)), left 4D finger means to age in three populations (European origin (**f**), African origin (**g**), Asian origin (**h**)).

**Figure 4:**
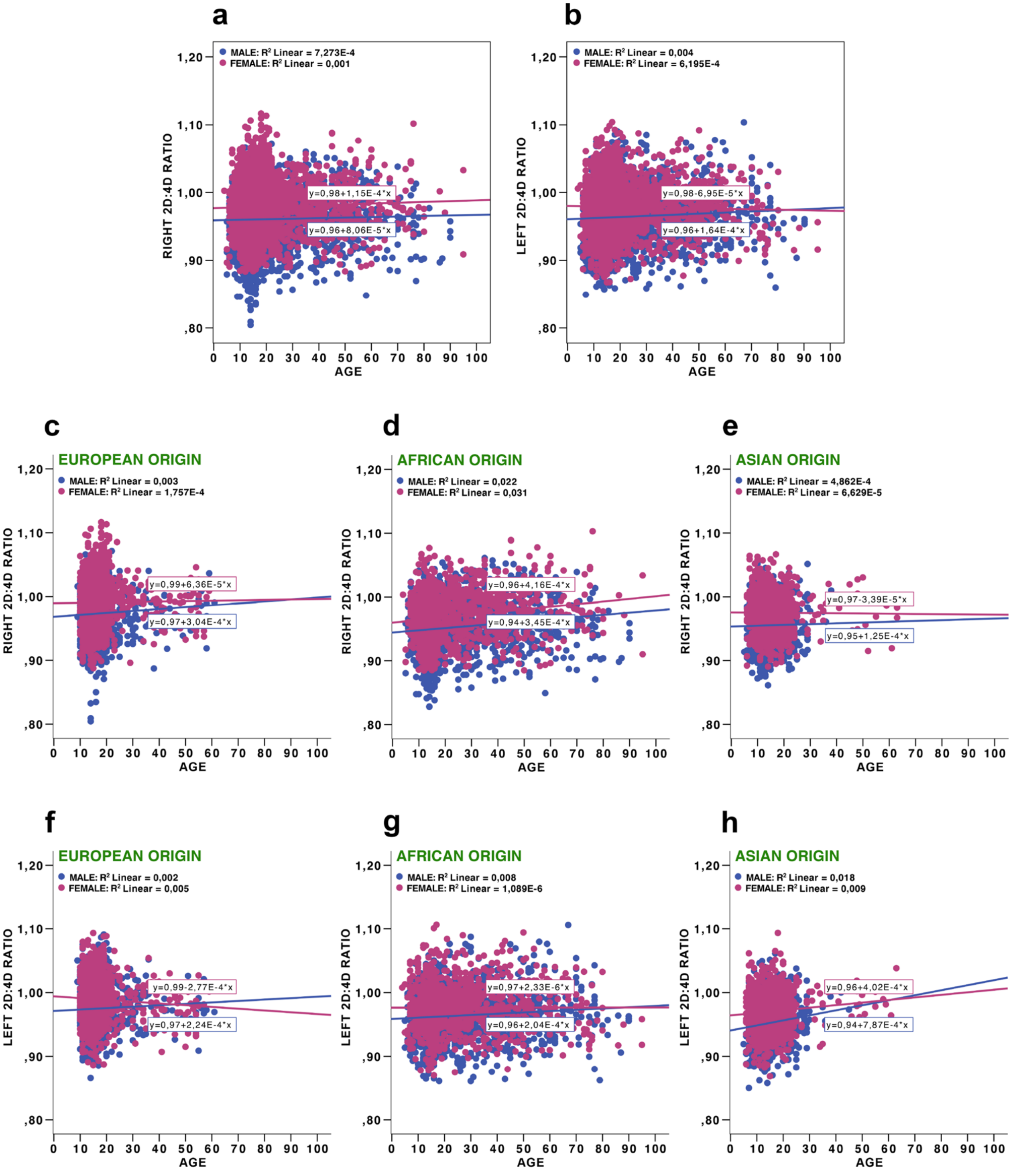
Right and left 2D:4D ratios to age: right 2D:4D ratio to age in total sample (**a**), left 2D:4D ratio to age in total sample (**b**), right 2D:4D ratios to age in three populations (European origin (**c**), African origin (**d**), Asian origin (**e**)), left 2D:4D ratios to age in three populations (European origin (**f**), African origin (**g**), Asian origin (**h**)).

The life trajectories of the second and fourth finger lengths, as well as 2D:4D on both hands, were tested in a set of linear regressions for the whole sample and separately for the three populations (Figs. 2, 3, 4).

Given the goals of this study, we were also interested in determining whether male fingers were always longer than female fingers, whether developmental trajectories for the two sexes look different, and whether these transformations may have affected the 2D:4D during periods of intensive growth and development. Thus, in the next step we focused on the subsample of four separate age cohort individuals with special emphases on prepubertal, pubertal, and young adults (see the ‘Procedure’ section). As mentioned earlier, the fourth age group was substantially underrepresented in the case of Europeans and Asians, and consequently, the results obtained in this case should be received with caution. The descriptive statistics for the 2D:4D ratio, mean finger length, and average finger length for each age cohort independently for the whole sample (Table 3), and separately for each population (in this case, the data for the right hand only were provided) (Supplementary Table 1) is provided. The latter was performed to reduce the number of tests and in accordance with the general assumption about more evident tendencies of androgenisation in the right than in the left hand^65–67^. In addition, there was a high correlation between the right and left hand second and fourth digit lengths in both sexes. T-tests for sex differences in second and fourth digit lengths, and 2D:4D in each of these cases, were also conducted (Table 3; Supplementary Table 1). For the first age cohort, the length of the second digit was longer in females than males, and the length of the fourth digit was not sexually dimorphic (both hands, total sample). In contrast, in the remaining three older age cohorts, both digits were longer in males than females (both hands, total sample). However, in all age cohorts, the 2D:4D ratios were lower in men than women. Additional information on developmental trajectories in second and fourth digit lengths, as well as 2D:4D ratios, are presented for prepubertal, pubertal, and young adults in supplementary figures (Supplementary Figs. 1–7).

**Table 3:** Sex differences in finger measurements and 2D:4D ratios for both hands in prepubertal, pubertal, young and older adults age cohorts.

Age groups	Parameters	Sex	*N*	Mean	SD	t	Df	P	95% confidence interval of the difference		Cohen’s d
									Lower	Upper	
13 years and younger	Right 2D finger	Male	1033	60.217	6.989	−2.898	2164	0.004	−1.445	−0.278	0.125
		Female	1133	61.079	6.839						
	Right 4D finger	Male	1032	62.884	7.082	0.861	2164	0.390	−0.328	0.842	0.037
		Female	1134	62.627	6.797						
	Right average finger length	Male	1032	61.550	6.940	−1.046	2163	0.296	−0.884	0.269	0.045
		Female	1133	61.858	6.729						
	Right 2D:4D ratio	Male	1032	0.958	0.036	−11.506	2116	9.3041E−30	−0.020	−0.014	0.486
		Female	1133	0.975	0.034						
	Left 2D finger	Male	937	60.668	7.245	−2.023	1912	0.043	−1.230	−0.020	0.092
		Female	977	61.328	7.028						
	Left 4D finger	Male	934	63.245	7.189	1.335	1910	0.182	−0.202	1.064	0.061
		Female	978	62.814	6.927						
	Left average finger length	Male	934	61.952	7.129	−0.358	1909	0.721	−0.745	0.515	0.017
		Female	977	62.067	6.899						
	Left 2D:4D ratio	Male	934	0.959	0.036	−10.886	1878	8.3729E−27	−0.020	−0.014	0.521
		Female	977	0.977	0.033						
14–18 years old	Right 2D finger	Male	1418	69.382	6.810	11.224	2543	1.4456E−28	2.057	2.928	0.242
		Female	1369	66.890	4.771						
	Right 4D finger	Male	1419	72.270	6.538	17.829	2556	4.1246E−67	3.393	4.231	0.673
		Female	1369	68.458	4.619						
	Right average finger length	Male	1416	70.832	6.535	14.892	2523	3.8712E−48	2.746	3.579	0.563
		Female	1368	67.670	4.520						
	Right 2D:4D ratio	Male	1416	0.960	0.037	−12.335	2783	4.4591E−34	−0.020	−0.015	0.459
		Female	1369	0.977	0.037						
	Left 2D finger	Male	1134	69.923	6.398	12.575	2057	5.3877E−35	2.480	3.397	0.402
		Female	1145	66.984	4.604						
	Left 4D finger	Male	1134	72.569	6.302	17.778	2063	6.6686E−66	3.648	4.553	0.691
		Female	1144	68.469	4.559						
	Left average finger length	Male	1134	71.246	6.226	15.544	2041	1.4072E−51	0.074	3.961	0.652
		Female	1144	67.729	4.413						
	Left 2D:4D ratio	Male	1134	0.964	0.034	−10.342	2276	1.5701E−24	−0.018	−0.012	0.428
		Female	1144	0.979	0.036						
19–30 years old	Right 2D finger	Male	774	70.942	4.917	14.251	1587	1.8781E−43	3.066	4.045	0.715
		Female	815	67.387	5.022						
	Right 4D finger	Male	772	73.667	5.149	21.093	1582	3.1447E−87	4.791	5.773	1.060
		Female	812	68.384	4.818						
	Right average finger length	Male	678	72.279	4.824	18.212	1578	2.0334E−67	3.912	4.856	0.811
		Female	812	67.895	4.743						
	Right 2D:4D ratio	Male	768	0.963	0.038	−11.921	1578	1.9734E−31	−0.026	−0.019	0.605
		Female	812	0.986	0.038						
	Left 2D finger	Male	661	70.341	5.270	9.508	1319	8.809E−21	2.127	3.233	0.531
		Female	665	67.662	4.989						
	Left 4D finger	Male	660	72.916	5.700	12.953	1306	3.4355E−36	3.279	4.450	0.712
		Female	663	69.051	5.136						
	Left average finger length	Male	659	71.632	5.313	11.589	1311	1.2304E−29	2.715	3.822	0.638
		Female	662	68.363	4.930						
	Left 2D:4D ratio	Male	659	0.966	0.038	−7.520	1300	1.0203E−13	−0.019	−0.011	0.388
		Female	662	0.980	0.033						
31 years and older	Right 2D finger	Male	535	70.640	5.122	11.380	972	2.9648E−28	3.136	4.443	0.732
		Female	439	66.852	5.230						
	Right 4D finger	Male	534	73.443	5.240	14.826	972	5.4974E−45	4.436	5.789	0.952
		Female	440	68.331	5.492						
	Right average finger length	Male	524	71.974	4.983	13.433	959	8.102E−38	3.775	5.067	
		Female	437	67.553	5.196						0.868
	Right 2D:4D ratio	Male	524	0.963	0.037	−7.419	959	2.5935E−13	−0.022	0.013	0.472
		Female	437	0.980	0.035						
	Left 2D finger	Male	542	69.814	5.938	4.134	988	0.000034	0.758	2.111	0.263
		Female	448	68.380	4.903						
	Left 4D finger	Male	533	72.284	6.112	5.826	969	7.7021E−9	1.430	2.883	0.373
		Female	443	70.127	5.446						
	Left average finger length	Male	529	71.013	5.878	5.088	969	4.3402E−7	1.093	2.466	0.326
		Female	442	69.233	5.020						
	Left 2D:4D ratio	Male	529	0.967	0.037	−3.555	969	0.000396	−0.013	−0.004	0.219
		Female	442	0.975	0.036						

Sex differences presented according to Student’s T test (*t* test statistics, *SD* std. deviation, *df* degrees of freedom, *p* statistical significance).

To test for possible differences in the effect of average finger lengths on 2D:4D in different periods of growth and development, we conducted the GLM ANCOVAs for separate age cohorts for the whole sample (Table 4), and separately for each population (Supplementary Table 2). The effect of sex on the right hand digit ratio was significant (medium effect size in the case of the youngest age cohort, and small effect sizes in the rest of the cases, total sample). Population was a significant predictor of digit ratio for the three younger age cohorts (total sample) (Table 4). Neither age nor average digit length were significant predictors of digit ratios in separate age cohorts (total sample and separate populations) (Table 4; Supplementary Table 2).

**Table 4:** Four-factor (sex, population, age, average finger length for the right hand) ANCOVA analyses for outcome variables the right 2D:4D ratio in prepubertal, pubertal, young and older adults age cohorts.

Age groups	Dependent variable	R^2^	df	Independent variables	F	P	η^2^
Until 13 years old	R2D:4D	0.121	1	Sex	160.934	1.2773E−35	0.069
			2	Population	64.317	7.3779E−28	0.056
			1	Age	0.620	0.431	0.000
			1	R average finger length	0.075	0.784	0.000
14–18 years old	R2D:4D	0.121	1	Sex	130.801	1.2461E−29	0.045
			2	Population	150.182	1.1683E−62	0.098
			1	Age	10.172	0.001	0.004
			1	R average finger length	0.000	0.995	0.000
19–30 years old	R2D:4D	0.121	1	Sex	77.009	4.339E−18	0.047
			2	Population	91.254	3.1842E−38	0.104
			1	Age	2.100	0.148	0.001
			1	R average finger length	5.639	0.018	0.004
31 years and older	R2D:4D	0.061	1	Sex	37.176	1.5652E−9	0.037
			2	Population	1.766	0.172	0.004
			1	Age	2.851	0.092	0.003
			1	R average finger length	0.317	0.574	0.0003

*R*^*2*^ R Squared, *df* degrees of freedom, *F* F test statistics, *p* statistical significance, *η*^*2*^ Partial Eta Squared effect size.

Height was used as another measure of allometric effect on 2D:4D for a limited sample from the European population within the 9 to 30 years age range. The GLM ANCOVA analyses were conducted for the right hand 2D:4D with outcome variable, sex, age, and height as predictors for the whole sample, as well as separately for prepubertal, pubertal, and young adult age cohorts. None of the tests revealed any significant height effect on 2D:4D (Table 5).

**Table 5:** The GLM ANCOVA three-factor (sex, age, height) analyses for outcome variables the right 2D:4D ratio in total sample (until 30 years old), and prepubertal, pubertal, and young age cohorts from European population.

Age groups	Dependent variable	R^2^	df	Independent variables	F	P	η^2^
Total sample	R2D:4D	0.064	1	Sex	135.445	1.736E−30	0.054
			1	Age	6.613	0.010	0.003
			1	Height	0.032	0.859	0.000
Until 13 years old	R2D:4D	0.077	1	Sex	56.125	2.063E−13	0.074
			1	Age	0.794	0.373	0.001
			1	Height	0.105	0.745	0.000
14–18 years old	R2D:4D	0.121	1	Sex	37.977	9.6081E−10	0.029
			1	Age	11.184	0.000849	0.009
			1	Height	1.182	0.277	0.001
19–30 years old	R2D:4D	0.121	1	Sex	20.786	0.000007	0.048
			1	Age	2.068	0.151	0.005
			1	Height	1.531	0.217	0.004

*R*^*2*^ R Squared, *df* degrees of freedom, *F* F test statistics, *p* statistical significance, *η*^*2*^ Partial Eta Squared effect size.

## Discussion

The main conclusion of our study is that 2D:4D ratios on the right and left hands were sexually dimorphic for the whole sample, as well as separately for all three tested populations. This was not the case with the second and fourth digits and their averages. The effect sizes, Cohen’s d of sex differences for the 2D:4Ds, as well as for the second and fourth digits, and averages for both digits ranged from small to medium. We demonstrate that for the whole sample, as well as for separate populations, every 0.9 cm increase in the second digit was related to a 1.0 cm increase in fourth digit. The digit lengths (second and fourth) increased substantially from childhood to adulthood, and there was a strong positive correlation between second and fourth digits across individuals. These findings are in accordance with those of earlier studies^43^. Sexual dimorphism in digit lengths, evident in human adults, was not observed in prepubertal children, which is again in line with previously reported data^48^.

The developmental allometry effects were tested in four age cohorts, with special emphasis on younger subsamples. This was conducted in accordance with the knowledge about the intensive growth of fingers in this period, as well as existing data on changes in growth patterns from childhood to young adulthood. The fourth age cohort (older adults) was substantially underrepresented and contained an insufficient number of individuals of European and Asian origin. Hence, we refrained from drawing specific conclusions for this age cohort.

For the whole sample, Cohen’s d for the 2D:4D ratios and digit lengths were of comparable sizes. In the case of separate prepubertal, pubertal, and young adult age cohorts, the situation was radically different. The data on sex differences in digit lengths in prepubertal and pubertal cohorts were in accordance with general expectations that females reach puberty considerably earlier than males^68–70^. On average, these results resulted in one to two-year differences^71^. Females in the prepubertal age cohort had significantly longer second digits and significantly higher 2D:4D ratios on both hands than males, whereas no sex differences for the fourth digits on both hands were found. For the prepubertal sample, the effect sizes for 2D:4D (right and left hand) were approximately 0.5 standard deviations, while for separate and average digits, the effect sizes were four times lower at the minimum.

Obvious population differences need to be considered. For prepubertal children in the African population, both the second and fourth digits were significantly longer in females, whereas for Europeans of the same age cohort, this was true only for the second digit; for Asians, no sex differences in digit length were present for prepubertal children. For the second age cohort, both fingers became significantly longer in males for Europeans and Asians, but were of equal lengths in males and females from the African sample. These differences suggest that respondents from African samples matured slower, and developmental processes in this population had different trajectories than their European and Asian peers. In young and older adults, both the second and fourth digits on both hands were significantly longer for males in all three populations. The 2D:4D ratio magnitude of sex differences essentially remained stable throughout ontogeny (in all four age cohorts) and of medium effect size. According to the logic of Lolli et al.^41,42^, the 2D:4D values would have to decrease with an increase in digit length, particularly in prepubertal and pubertal samples. In reality, the 2D:4Ds were remarkably stable with age, despite the increase of second and fourth digit length during ontogeny and the reversions in finger lengths in males and females that occurred during puberty.

Our results based on cross-sectional data are in line with other cross-sectional data, as well as with longitudinal studies^30^. Our data simultaneously revealed some population-specific variations in ontogenetic trajectories. In particular, in the African sample, female digits remained longer than male digits until 15–16 years of age (mid-adolescence), while in European and Asian samples, finger lengths were inversed before the age of 14 and remained longer in males than in females in all older ages. The differences obtained for Africans may be caused by specific life history trajectories with slower maturation, resulting from a mixture of environmental and social stressors, including malnutrition, a high risk of infections, and limited access to modern medical assistance in rural African populations^72–76^.

The GLM ANCOVA tests conducted in our study demonstrated the significant effects of sex and population origin (medium size), and a small effect size for age as predictors of 2D:4D. However, the average finger length was not a significant predictor for the right 2D:4D in accordance with recently reported data for adult Hadza males by other authors^77^. The effect of height as another potential marker of allometry on 2D:4D has not been detected in a limited sample of respondents until the age of 30; however, the data on height were tested for Europeans only. We do not know if the same peculiarities will be present in other large world populations as well as in older age cohorts. More data in this respect will be needed in the future to confirm these results.

Many previous studies have demonstrated that population/ethnic origin may be an important predictor for the 2D:4D ratio^36,37,78–82^, and our data support these conclusions. Along with numerous environmental factors, the heritability factor needs to be considered in this respect^83–85^. In particular, twin studies provide an estimate of approximately 60%^76^. Another support in favour of the inheritance of digit ratios has recently been presented by Chuvashian studies^85,86^. The clear familial aggregation of 2D:4D ratio variation in the Chuvashians, with significant parent–offspring and sibling correlations, was unrelated to common environmental effects. Hence, along with the various environmental and socio-cultural factors, certain genetic effects also need to be considered and tested with more care in the future.

In this study, we refrained from analysing the right-left difference in 2D:4D (D[R-L]). This was done deliberately, not only to limit the amount of information for analysis, but also for the following reasons: 1. the lack of studies on the validity of this marker; 2. using asymmetry in digits two and/or four may cause biases in the associations between asymmetry and digit ratios^87^; and 3. currently expressed concerns regarding the utility of D[R-L] as an indicator of prenatal androgen exposure^22^.

In summary, our data suggest that there is no reason to reject the sexual dimorphism of 2D:4D associated with both prenatal and postnatal factors. Hence, we should not throw the baby out with the bathwater. The sex differences in second and fourth digit lengths were not stable within ontogeny, and even reversed in adulthood, whereas 2D:4D ratios remained unchanged since six years of age. The ontogenetic transformations in finger lengths in boys and girls do not make the sex effect on 2D:4D less statistically significant. The effect size of sex was higher than the average digit length in all cases, and height (in the case of Europeans, less than 30 years of age). The theory according to which the sex difference in 2D:4D has been driven by the sex difference in digit length may have arisen from a misunderstanding and incorrect assumption ignoring the human growth pattern trajectory. The stability of 2D:4D may be an example of homeostasis of form, and our data completely support J. Manning and B. Fink’s idea^30^. Another important conclusion is that the degree to which androgenisation (2D:4D being a potential proxy) affects particular behaviour or morpho-physiological conditions may be population- and situation-specific and culturally mediated. Our data, along with currently accumulated information from different world populations, call for treating the allometric effect on 2D:4D with caution. It is worth stressing the importance of differentiation between the static and developmental allometry effects, and the necessity of considering as many factors as possible (genetics, particularly population origin, environmental and social factors affecting maturation, urbanisation effects, etc.) while searching for explanations of 2D:4D sex differences^30,88^. Populations currently undergoing rapid transformations due to global and local changes must be treated with special care. Representatives of different age cohorts may differ in their maturation patterns, particularly the timing and duration of growth morphological changes. Due to the small to medium effect sizes that are usually obtained, studies using 2D:4D may need to consider very large sample sizes in order to be of practical use^89^. However, 2D:4D remains a useful measure of sexual dimorphism for anthropologists. Along with a set of other sexually dimorphic traits, it may be used in projects dealing with ecological and socio-cultural transformations in growth and development in contemporary representatives from large- and small-scale societies.

## Supplementary Information

Supplementary Information.

Supplementary Figure legends.

Supplementary Figure 1.

Supplementary Figure 2.

Supplementary Figure 3.

Supplementary Figure 4.

Supplementary Figure 5.

Supplementary Figure 6.

Supplementary Figure 7.

Supplementary Table 1.

## Data Availability

The data produced and processed in this study are included in the published article and supplementary files. The datasets were acquired from the corresponding author for appropriate purposes.
