# Supplementary material for: Sex, population origin, age and average digit length as predictors of digit ratio in three large world populations
Source: Sci Rep. 2021 Apr 14;11:8157. doi: 10.1038/s41598-021-87394-6 (PMC8046776; doi:10.1038/s41598-021-87394-6)
Supplement: Supplementary file 1 — Supplementary Information. [file 41598_2021_87394_MOESM1_ESM.docx]

**Sex, population origin, age and average digit length as predictors of digit ratio in the three large world populations**

Butovskaya M.^1,2,3 *^, Burkova V.^1,2^, Apalkova J.^1^, Dronova D.^1^, Rostovtseva V.^1^, Karelin D.^4^, Mkrtchyan R.^5^, Negasheva M.^6^, Batsevich V.^6^

1. Institute of Ethnology and Anthropology, RAS, Moscow, Russia
2. National Research University Higher School of Economics, Moscow, Russia
3. Russian State University for the Humanities
4. Institute of Geography, RAS, Moscow Russia
5. Yerevan State University, Yerevan, Armenia
6. Moscow State University, Moscow, Russia

Butovskaya Marina

Burkova Valentina

Apalkova Julia

Dronova Daria

Rostovtseva Victoria

Karelin Dmitry

Mkrtchyan Ruzan

Negasheva Marina

Batsevich Valery

** Corresponding author* at: Institute of Ethnology and Anthropology, Russian Academy of Sciences, Leninsky pr., 32a, Moscow 119991, Russia. E-mail address: marina.butovskaya@gmail.com (M. Butovskaya).
