# Supplementary material for: Sex, population origin, age and average digit length as predictors of digit ratio in three large world populations
Source: Sci Rep. 2021 Apr 14;11:8157. doi: 10.1038/s41598-021-87394-6 (PMC8046776; doi:10.1038/s41598-021-87394-6)
Supplement: Supplementary file 2 — Supplementary Figure legends. [file 41598_2021_87394_MOESM2_ESM.docx]

**FIGURE LEGENDS IN SUPPLEMENTARY**

**Figure 1.** Ratio right 2D finger means to right 4D finger means in three age cohorts:

age until 13 years in total sample (panel **a**), in three populations – European origin (panel **b**), African origin (panel **c**), Asian origin (panel **d**);

age 14-18 years old in total sample (panel **e**), in three populations – European origin (panel **f**), African origin (panel **g**), Asian origin (panel **h**);

age 19-30 years old in total sample (panel **i**), in three populations – European origin (panel **j**), African origin (panel **k**), Asian origin (panel **l**).

**Figure 2.** Right 2D:4D ratio, right 2D and 4D finger means to age until 13 years old:

right 2D:4D ratio to age in total sample (panel **a**), in three populations – European origin (panel **b**), African origin (panel **c**), Asian origin (panel **d**);

right 2D finger means to age in total sample (panel **e**), in three populations – European origin (panel **f**), African origin (panel **g**), Asian origin (panel **h**);

right 4D finger means to age in total sample (panel **i**), in three populations – European origin (panel **j**), African origin (panel **k**), Asian origin (panel **l**).

**Figure 3.** Right 2D:4D ratio, right 2D and 4D finger means to age in 14-18 years old:

right 2D:4D ratio to age in total sample (panel **a**), in three populations – European origin (panel **b**), African origin (panel **c**), Asian origin (panel **d**);

right 2D finger means to age in total sample (panel **e**), in three populations – European origin (panel **f**), African origin (panel **g**), Asian origin (panel **h**);

right 4D finger means to age in total sample (panel **i**), in three populations – European origin (panel **j**), African origin (panel **k**), Asian origin (panel **l**).

**Figure 4.** Right 2D:4D ratio, right 2D and 4D finger means to age in 19-30 years old:

right 2D:4D ratio to age in total sample (panel **a**), in three populations – European origin (panel **b**), African origin (panel **c**), Asian origin (panel **d**);

right 2D finger means to age in total sample (panel **e**), in three populations – European origin (panel **f**), African origin (panel **g**), Asian origin (panel **h**);

right 4D finger means to age in total sample (panel **i**), in three populations – European origin (panel **j**), African origin (panel **k**), Asian origin (panel **l**).

**Figure 5.** Right 2D:4D ratio to age in total sample of European population until 30 years old (panel **a**), and separately for three age cohorts – until 13 years old (panel **b**), 14-18 years old (panel **c**), 19-30 years old (panel **d**).

**Figure 6.** Right 2D and 4D means to height (in meter) of European population until 30 years old in three age groups: right 2D means to height until 13 years old (panel **a**), 14-18 years old (panel **b**), 19-30 years old (panel **c**); right 4D means to height until 13 years old (panel **d**), 14-18 years old (panel **e**), 19-30 years old (panel **f**).

**Figure 7.** Height (in meter) to age in total sample of European population until 30 years old (panel **a**), and separately for three age cohorts – until 13 years old (panel **b**), 14-18 years old (panel **c**), 19-30 years old (panel **d**).
