# Supplementary figures and images for: Sex, population origin, age and average digit length as predictors of digit ratio in three large world populations
Source: Sci Rep. 2021 Apr 14;11:8157. doi: 10.1038/s41598-021-87394-6 (PMC8046776; doi:10.1038/s41598-021-87394-6)

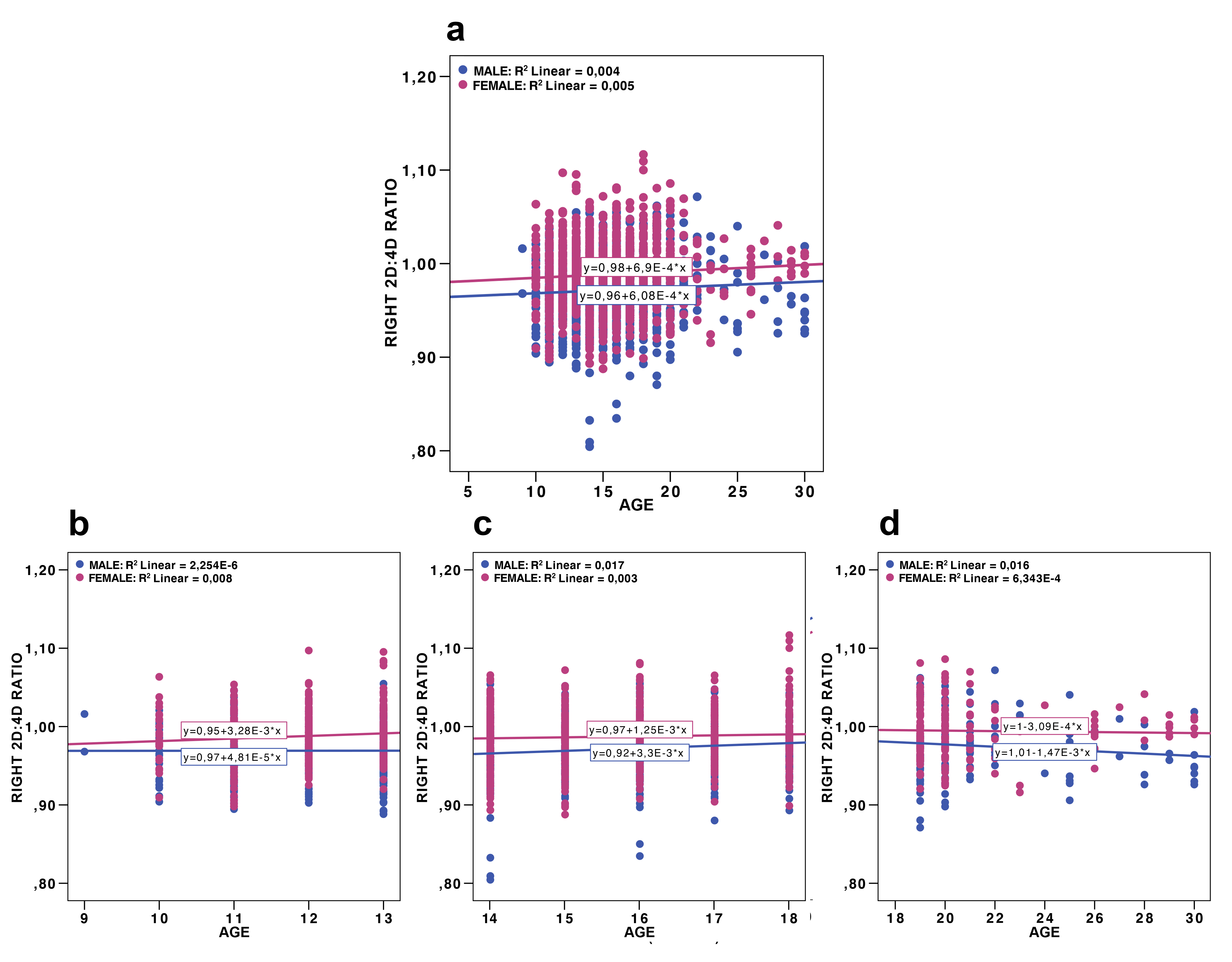

Supplement: Supplementary file 7 — Supplementary Figure 5. [file 41598_2021_87394_MOESM7_ESM.tif]

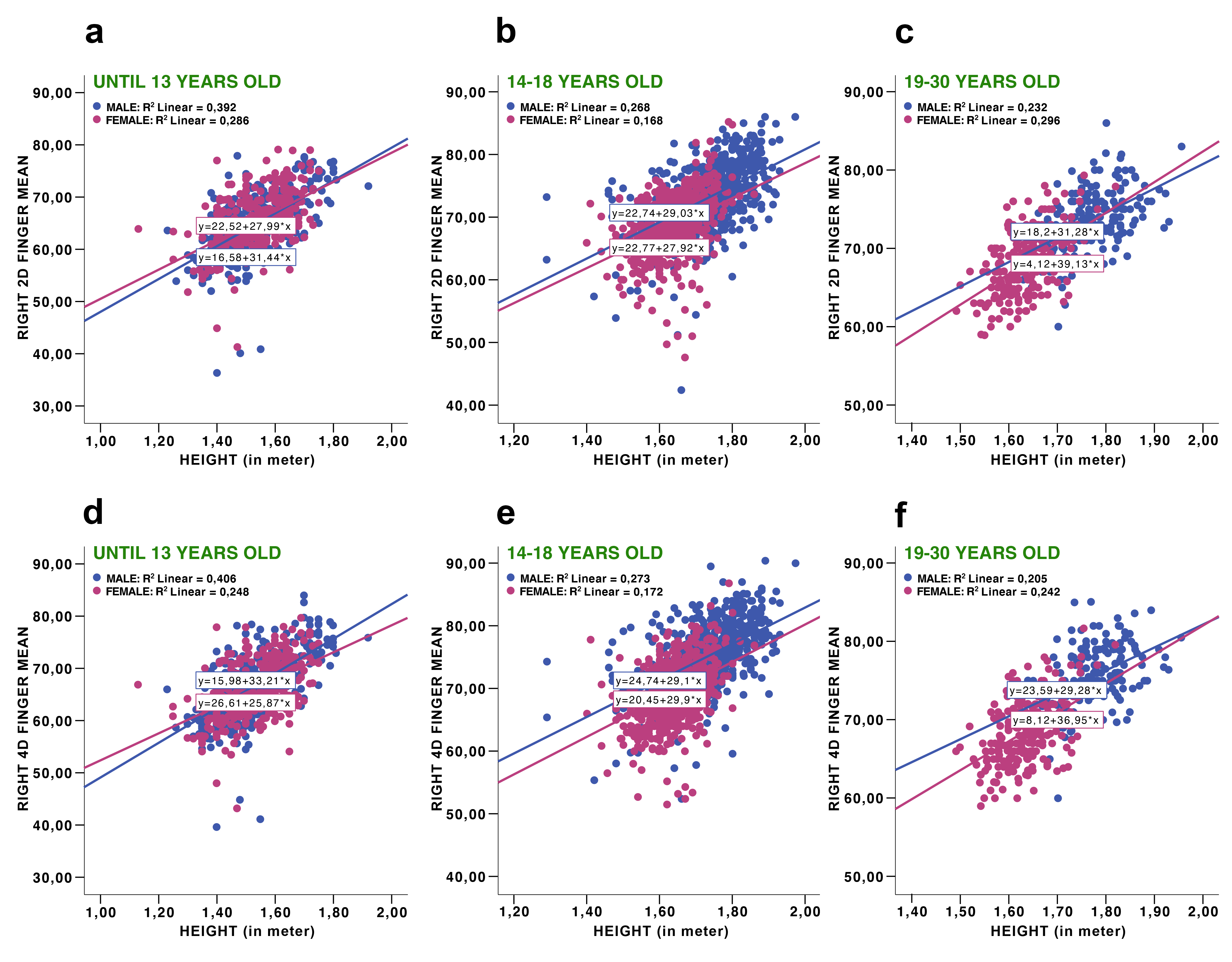

Supplement: Supplementary file 8 — Supplementary Figure 6. [file 41598_2021_87394_MOESM8_ESM.tif]

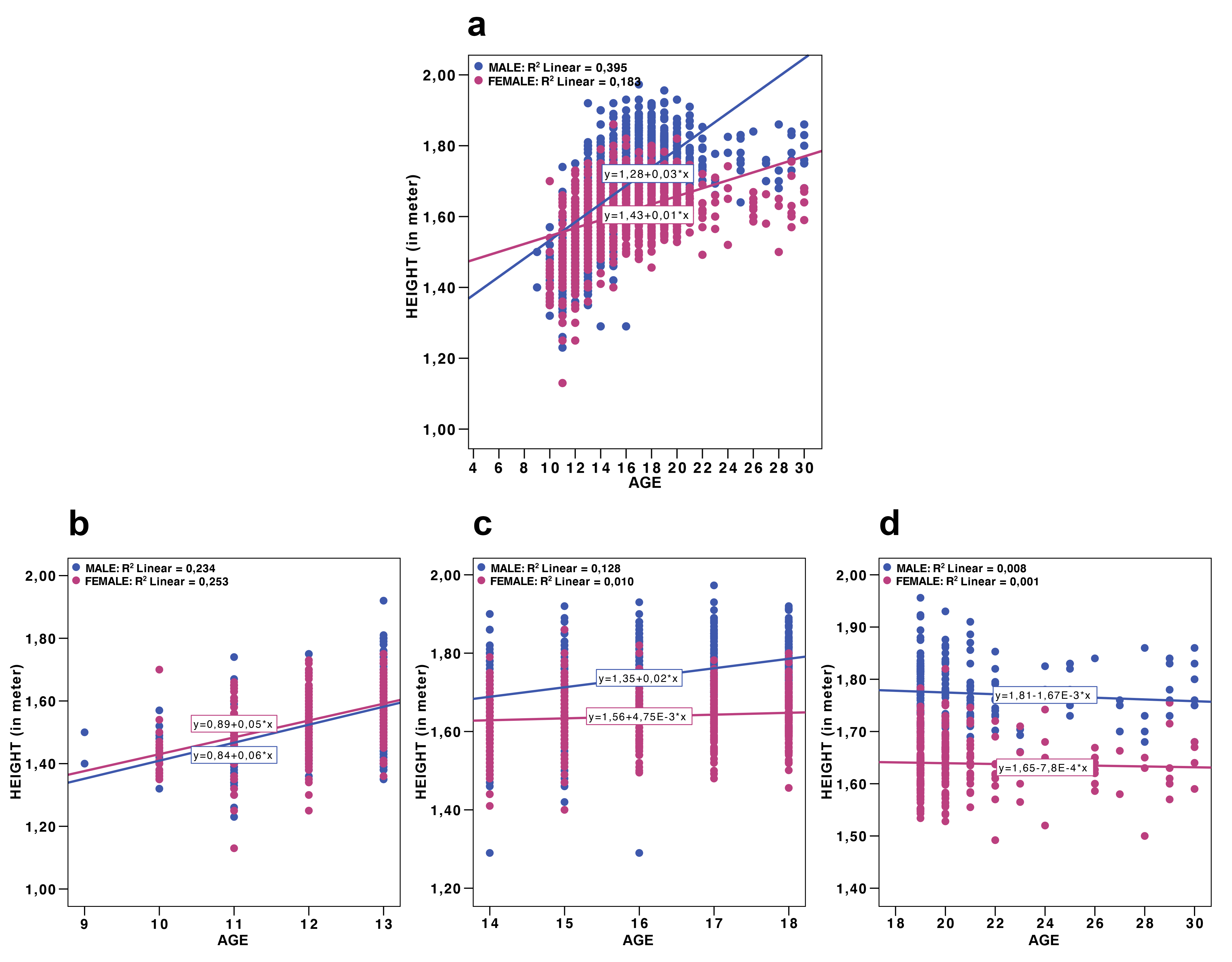

Supplement: Supplementary file 9 — Supplementary Figure 7. [file 41598_2021_87394_MOESM9_ESM.tif]
