# Supplementary material for: Sex, population origin, age and average digit length as predictors of digit ratio in three large world populations
Source: Sci Rep. 2021 Apr 14;11:8157. doi: 10.1038/s41598-021-87394-6 (PMC8046776; doi:10.1038/s41598-021-87394-6)
Supplement: Supplementary file 10 — Supplementary Table 1. [file 41598_2021_87394_MOESM10_ESM.docx]

**SUPPLEMENTARY**

**Table 1. Sex differences in finger measurements and 2D:4D ratios on the right hand in prepubertal, pubertal, young and older adults age cohorts in Europeans, Africans and Asians**

| **Population** | **Age groups** | **Parameters** | **Sex** | ***N*** | **Mean** | **SD** | **t** | **Df** | **P** | **95% Confidence Interval of the Difference** | | **Cohen’s d** |
| --- | --- | --- | --- | --- | --- | --- | --- | --- | --- | --- | --- | --- |
|  |  |  |  |  |  |  |  |  |  | **Lower** | **Upper** |  |
| European origin | **13 years old and younger** | Right 2D finger | male | 413 | 64.397 | 5.924 | -2.474 | 797 | 0.015 | -1.748 | -0.201 | 0.175 |
|  |  |  | female | 389 | 65.372 | 5.227 |  |  |  |  |  |  |
|  |  | Right 4D finger | male | 413 | 66.517 | 6.106 | 0.541 | 792 | 0.589 | -0.568 | 1.000 | 0.038 |
|  |  |  | female | 389 | 66.301 | 5.183 |  |  |  |  |  |  |
|  |  | Right average finger length | male | 413 | 65.458 | 5.908 | -0.976 | 794 | 0.329 | -1.142 | 0.383 | 0.069 |
|  |  |  | female | 389 | 65.837 | 5.084 |  |  |  |  |  |  |
|  |  | Right 2D:4D ratio | male | 413 | 0.969 | 0.033 | -7.501 | 800 | 1.6909E-13 | -0.022 | -0.013 | 0.507 |
|  |  |  | female | 389 | 0.986 | 0.038 |  |  |  |  |  |  |
|  | **14-18 years old** | Right 2D finger | male | 698 | 73.042 | 5.122 | 17.584 | 1387 | 1.2136E-62 | 3.988 | 4.990 | 0.923 |
|  |  |  | female | 736 | 68.553 | 4.506 |  |  |  |  |  |  |
|  |  | Right 4D finger | male | 697 | 75.072 | 5.223 | 21.833 | 1385 | 4.7299E-91 | 5.176 | 6.197 | 1.168 |
|  |  |  | female | 735 | 69.385 | 4.593 |  |  |  |  |  |  |
|  |  | Right average finger length | male | 697 | 74.055 | 5.004 | 30.436 | 1383 | 2.5307E-81 | 4.602 | 5.579 | 1.082 |
|  |  |  | female | 735 | 68.964 | 4.382 |  |  |  |  |  |  |
|  |  | Right 2D:4D ratio | male | 697 | 0.973 | 0.035 | -8.147 | 1430 | 8.0432E-16 | -0.019 | -0.011 | 0.457 |
|  |  |  | female | 735 | 0.989 | 0.035 |  |  |  |  |  |  |
|  | **19-30 years old** | Right 2D finger | male | 276 | 73.034 | 4.383 | 9.637 | 644 | 1.2735E-20 | 2.791 | 4.220 | 0.771 |
|  |  |  | female | 370 | 69.528 | 4.711 |  |  |  |  |  |  |
|  |  | Right 4D finger | male | 274 | 74.595 | 4.868 | 13.340 | 641 | 5.3134E-36 | 4.381 | 5.894 | 1.062 |
|  |  |  | female | 369 | 69.458 | 4.780 |  |  |  |  |  |  |
|  |  | Right average finger length | male | 273 | 73.780 | 4.392 | 11.903 | 640 | 1.1931E-29 | 3.576 | 4.989 | 0.961 |
|  |  |  | female | 369 | 69.496 | 4.591 |  |  |  |  |  |  |
|  |  | Right 2D:4D ratio | male | 273 | 0.980 | 0.038 | -7.605 | 640 | 1.0211E-13 | -0.028 | -0.017 | 0.594 |
|  |  |  | female | 369 | 1.002 | 0.036 |  |  |  |  |  |  |
|  | **31 years and older** | Right 2D finger | male | 58 | 75.052 | 4.138 | 6.465 | 137 | 1.6474E-9 | 3.027 | 5.695 | 1.103 |
|  |  |  | female | 81 | 70.690 | 3.759 |  |  |  |  |  |  |
|  |  | Right 4D finger | male | 57 | 77.689 | 4.838 | 7.036 | 137 | 8.6877E-11 | 3.930 | 7.004 | 1.199 |
|  |  |  | female | 82 | 72.221 | 4.263 |  |  |  |  |  |  |
|  |  | Right average finger length | male | 51 | 76.189 | 4.363 | 6.738 | 129 | 4.8002E-10 | 3.422 | 6.267 | 1.188 |
|  |  |  | female | 80 | 71.345 | 3.773 |  |  |  |  |  |  |
|  |  | Right 2D:4D ratio | male | 51 | 0.973 | 0.030 | -1.792 | 129 | 0.075 | -0.029 | 0.001 | 0.345 |
|  |  |  | female | 80 | 0.983 | 0.028 |  |  |  |  |  |  |
| African origin | **13 years old and younger** | Right 2D finger | male | 286 | 55.699 | 7.725 | -4.920 | 651 | 0.000001 | -9.400 | -1.460 | 0.383 |
|  |  |  | female | 370 | 58.130 | 6.918 |  |  |  |  |  |  |
|  |  | Right 4D finger | male | 286 | 58.889 | 6.360 | -2.692 | 644 | 0.007 | -2.484 | -0.388 | 0.210 |
|  |  |  | female | 370 | 60.326 | 7.283 |  |  |  |  |  |  |
|  |  | Right average finger length | male | 286 | 57.294 | 5.925 | -3.822 | 649 | 0.000145 | -2.927 | -0.940 | 0.298 |
|  |  |  | female | 380 | 59.228 | 7.018 |  |  |  |  |  |  |
|  |  | Right 2D:4D ratio | male | 286 | 0.947 | 0.039 | -5.983 | 654 | 3.6006E-9 | -0.023 | -0.012 | 0.459 |
|  |  |  | female | 370 | 0.964 | 0.035 |  |  |  |  |  |  |
|  | **14-18 years old** | Right 2D finger | male | 462 | 64.024 | 6.506 | -0.751 | 812 | 0.453 | -1.062 | 0.474 | 0.052 |
|  |  |  | female | 356 | 64.318 | 4.678 |  |  |  |  |  |  |
|  |  | Right 4D finger | male | 462 | 67.917 | 6.850 | 1.890 | 810 | 0.059 | -0.030 | 1.577 | 0.130 |
|  |  |  | female | 356 | 67.143 | 4.844 |  |  |  |  |  |  |
|  |  | Right average finger length | male | 461 | 65.982 | 6.549 | 0.643 | 808 | 0.521 | -0.516 | 1.018 | 0.044 |
|  |  |  | female | 356 | 65.731 | 4.602 |  |  |  |  |  |  |
|  |  | Right 2D:4D ratio | male | 461 | 0.944 | 0.037 | -5.831 | 815 | 7.9488E-9 | -0.020 | -0.010 | 0.417 |
|  |  |  | female | 356 | 0.959 | 0.035 |  |  |  |  |  |  |
|  | **19-30 years old** | Right 2D finger | male | 293 | 68.898 | 5.144 | 7.640 | 534 | 1.0111E-13 | 2.497 | 4.225 | 0.664 |
|  |  |  | female | 243 | 65.537 | 4.980 |  |  |  |  |  |  |
|  |  | Right 4D finger | male | 293 | 72.261 | 5.765 | 10.230 | 534 | 1.4982E-22 | 3.936 | 5.807 | 0.892 |
|  |  |  | female | 243 | 67.389 | 5.135 |  |  |  |  |  |  |
|  |  | Right average finger length | male | 292 | 70.568 | 5.292 | 9.235 | 533 | 6.088E-19 | 3.232 | 4.978 | 0.805 |
|  |  |  | female | 243 | 66.463 | 4.902 |  |  |  |  |  |  |
|  |  | Right 2D:4D ratio | male | 292 | 0.955 | 0.037 | -5.831 | 533 | 9.5661E-9 | -0.025 | -0.012 | 0.493 |
|  |  |  | female | 243 | 0.973 | 0.036 |  |  |  |  |  |  |
|  | **31 years and older** | Right 2D finger | male | 470 | 70.111 | 4.971 | 11.594 | 797 | 7.8435E-29 | 3.511 | 4.943 | 0.830 |
|  |  |  | female | 329 | 65.884 | 5.212 |  |  |  |  |  |  |
|  |  | Right 4D finger | male | 471 | 72.941 | 5.073 | 14.963 | 798 | 8.4382E-45 | 4.883 | 6.358 | 1.068 |
|  |  |  | female | 329 | 67.321 | 5.442 |  |  |  |  |  |  |
|  |  | Right average finger length | male | 467 | 71.521 | 4.841 | 13.726 | 793 | 1.269E-38 | 4.223 | 5.632 | 0.983 |
|  |  |  | female | 328 | 66.594 | 5.178 |  |  |  |  |  |  |
|  |  | Right 2D:4D ratio | male | 467 | 0.961 | 0.037 | -6.827 | 793 | 1.7227E-11 | -0.024 | -0.013 | 0.514 |
|  |  |  | female | 328 | 0.980 | 0.037 |  |  |  |  |  |  |
| Asian origin | **13 years old and younger** | Right 2D finger | male | 334 | 58.917 | 6.244 | -1.333 | 706 | 0.183 | -1.517 | 0.290 | 0.100 |
|  |  |  | female | 374 | 59.530 | 5.995 |  |  |  |  |  |  |
|  |  | Right 4D finger | male | 333 | 61.808 | 6.590 | 1.504 | 706 | 0.133 | -0.220 | 1.664 | 0.113 |
|  |  |  | female | 375 | 61.086 | 6.172 |  |  |  |  |  |  |
|  |  | Right average finger length | male | 333 | 60.360 | 6.332 | 0.083 | 705 | 0.934 | -0.873 | 0.950 | 0.060 |
|  |  |  | female | 374 | 60.322 | 6.005 |  |  |  |  |  |  |
|  |  | Right 2D:4D ratio | male | 333 | 0.954 | 0.033 | -8.642 | 673 | 4.0265E-17 | -0.026 | -0.016 | 0.666 |
|  |  |  | female | 374 | 0.975 | 0.030 |  |  |  |  |  |  |
|  | **14-18 years old** | Right 2D finger | male | 258 | 69.077 | 4.531 | 9.225 | 495 | 8.2483E-19 | 2.598 | 4.004 | 0.801 |
|  |  |  | female | 277 | 65.776 | 3.665 |  |  |  |  |  |  |
|  |  | Right 4D finger | male | 260 | 72.492 | 4.716 | 12.975 | 497 | 2.3326E-33 | 4.077 | 5.532 | 1.124 |
|  |  |  | female | 278 | 67.688 | 3.785 |  |  |  |  |  |  |
|  |  | Right average finger length | male | 258 | 70.795 | 4.491 | 11.548 | 490 | 1.8902E-27 | 3.374 | 4.758 | 1.004 |
|  |  |  | female | 277 | 66.729 | 3.561 |  |  |  |  |  |  |
|  |  | Right 2D:4D ratio | male | 258 | 0.953 | 0.030 | -9.001 | 534 | 7.6823E-12 | -0.024 | -0.014 | 0.613 |
|  |  |  | female | 278 | 0.972 | 0.032 |  |  |  |  |  |  |
|  | **19-30 years old** | Right 2D finger | male | 205 | 71.048 | 3.990 | 13.518 | 405 | 1.259E-34 | 4.579 | 6.138 | 1.340 |
|  |  |  | female | 202 | 65.689 | 4.008 |  |  |  |  |  |  |
|  |  | Right 4D finger | male | 205 | 74.435 | 4.040 | 17.079 | 403 | 1.3556E-49 | 6.037 | 7.607 | 1.698 |
|  |  |  | female | 200 | 67.612 | 3.997 |  |  |  |  |  |  |
|  |  | Right average finger length | male | 203 | 72.723 | 3.817 | 15.848 | 401 | 2.8849E-44 | 5.293 | 6.792 | 1.579 |
|  |  |  | female | 200 | 66.680 | 3.837 |  |  |  |  |  |  |
|  |  | Right 2D:4D ratio | male | 203 | 0.955 | 0.033 | -5.851 | 401 | 1.0147E-8 | -0.024 | -0.012 | 0.571 |
|  |  |  | female | 200 | 0.973 | 0.030 |  |  |  |  |  |  |
|  | **31 years and older** | Right 2D finger | male | 7 | 69.687 | 5.405 | 1.445 | 34 | 0.157 | -1.047 | 6.206 | 0.545 |
|  |  |  | female | 29 | 67.108 | 3.942 |  |  |  |  |  |  |
|  |  | Right 4D finger | male | 6 | 72.464 | 3.898 | 2.011 | 33 | 0.053 | -0.438 | 7.403 | 0.918 |
|  |  |  | female | 29 | 68.785 | 4.112 |  |  |  |  |  |  |
|  |  | Right average finger length | male | 6 | 71.454 | 4.661 | 1.949 | 33 | 0.060 | -0.154 | 7.178 | 0.818 |
|  |  |  | female | 29 | 67.946 | 3.885 |  |  |  |  |  |  |
|  |  | Right 2D:4D ratio | male | 6 | 0.971 | 0.029 | -0.375 | 33 | 0.710 | -0.032 | 0.022 | 0,169 |
|  |  |  | female | 29 | 0.976 | 0.030 |  |  |  |  |  |  |

Sex differences presented according to Student’s T test (t—test statistics, SD – Std. Deviation, df – degrees of freedom, p – statistical significance)

**Table 2. Three-factor (sex, age, average finger length for the hand) ANCOVA analyses for outcome variables right 2D:4D ratio in prepubertal, pubertal, young and older adults age cohorts in Europeans, Africans and Asians**

| Age groups | Population | Dependent variable | R^2^ | Df | Independent variables | F | P | η^2^ |
| --- | --- | --- | --- | --- | --- | --- | --- | --- |
| Until 13 years old | European origin | R2D:4D | 0.067 | 1  1  1 | Sex | 56.003 | 1.9132E-13 | 0.066 |
|  |  |  |  |  | Age | 0.471 | 0.493 | 0.001 |
|  |  |  |  |  | R average finger length | 0.318 | 0.573 | 0.000 |
|  | African origin | R2D:4D | 0.070 | 1  1  1 | Sex | 38.694 | 8.8633E-10 | 0.056 |
|  |  |  |  |  | Age | 0.000 | 0.997 | 0.000 |
|  |  |  |  |  | R average finger length | 2.753 | 0.098 | 0.004 |
|  | Asian origin | R2D:4D | 0.089 | 1  1  1 | Sex | 73.993 | 5.0873E-17 | 0.095 |
|  |  |  |  |  | Age | 1.948 | 0.163 | 0.003 |
|  |  |  |  |  | R average finger length | 0.934 | 0.334 | 0.001 |
| 14-18 years old | European origin | R2D:4D | 0.060 | 1  1  1 | Sex | 51.850 | 9.6526E-13 | 0.035 |
|  |  |  |  |  | Age | 16.124 | 0.000062 | 0.011 |
|  |  |  |  |  | R average finger length | 0.058 | 0.810 | 0.000 |
|  | African origin | R2D:4D | 0.070 | 1  1  1 | Sex | 34.317 | 6.7971E-9 | 0.041 |
|  |  |  |  |  | Age | 0.253 | 0.615 | 0.000 |
|  |  |  |  |  | R average finger length | 0.252 | 0.616 | 0.000 |
|  | Asian origin | R2D:4D | 0.089 | 1  1  1 | Sex | 40.844 | 3.6113E-10 | 0.071 |
|  |  |  |  |  | Age | 0.105 | 0.746 | 0.000 |
|  |  |  |  |  | R average finger length | 0.022 | 0.881 | 0.000 |
| 19-30 years old | European origin | R2D:4D | 0.060 | 1  1  1 | Sex | 33.048 | 1.3943E-8 | 0.049 |
|  |  |  |  |  | Age | 9.378 | 0.002 | 0.014 |
|  |  |  |  |  | R average finger length | 4.507 | 0.034 | 0.007 |
|  | African origin | R2D:4D | 0.070 | 1  1  1 | Sex | 22.423 | 0.000003 | 0.041 |
|  |  |  |  |  | Age | 0.004 | 0.952 | 0.000 |
|  |  |  |  |  | R average finger length | 3.415 | 0.065 | 0.006 |
|  | Asian origin | R2D:4D | 0.089 | 1  1  1 | Sex | 26.730 | 3.7083E-7 | 0.063 |
|  |  |  |  |  | Age | 0.777 | 0.379 | 0.003 |
|  |  |  |  |  | R average finger length | 0.574 | 0.449 | 0.001 |
| 31 years and older | European origin | R2D:4D | 0.061 | 1  1  1 | Sex | 1.022 | 0.314 | 0.008 |
|  |  |  |  |  | Age | 0.071 | 0.791 | 0.001 |
|  |  |  |  |  | R average finger length | 1.013 | 0.316 | 0.008 |
|  | African origin | R2D:4D | 0.061 | 1  1  1 | Sex | 35.609 | 3.6374E-9 | 0.043 |
|  |  |  |  |  | Age | 2.180 | 0.140 | 0.003 |
|  |  |  |  |  | R average finger length | 0.151 | 0.697 | 0.000 |
|  | Asian origin | R2D:4D | 0.061 | 1  1  1 | Sex | 0.349 | 0.559 | 0.011 |
|  |  |  |  |  | Age | 0.549 | 0.464 | 0.017 |
|  |  |  |  |  | R average finger length | 0.332 | 0.569 | 0.011 |

R^2^ – R Squared, df – degrees of freedom, F – F test statistics, p – statistical significance, η^2^ - Partial Eta Squared effect size
